# Necrotizing Enterocolitis in Moderate Preterm Infants

**DOI:** 10.1155/2018/4126245

**Published:** 2018-10-10

**Authors:** Jayasree Nair, Rachel Longendyke, Satyan Lakshminrusimha

**Affiliations:** ^1^Department of Pediatrics, Jacobs School of Medicine and Biomedical Sciences, University at Buffalo, Buffalo, NY 14203, USA; ^2^Department of Pediatrics, University of California, Davis, Sacramento, CA 95817, USA

## Abstract

Necrotizing enterocolitis (NEC) is a devastating morbidity usually seen in preterm infants, with extremely preterm neonates (EPT ≤28 weeks) considered at highest risk. Moderately preterm infants (MPT 28–34 weeks) constitute a large percentage of NICU admissions. In our retrospective data analysis of NEC in a single regional perinatal center, NEC was observed in 10% of extremely EPT and 7% of MPT, but only 0.7% of late-preterm/term admissions. There was an inverse relationship between postnatal age at onset of NEC and gestational age at birth. Among MPT infants with NEC, maternal hypertensive disorders (29%) and small for gestational age (SGA-15%) were more common than in EPT infants (11.6 and 4.6%, resp.). Congenital gastrointestinal anomalies were common among late preterm/term infants with NEC. SGA MPT infants born to mothers with hypertensive disorders are particularly at risk and should be closely monitored for signs of NEC. Identifying risk factors specific to each gestational age may help clinicians to tailor interventions to prevent NEC.

## 1. Introduction

Necrotizing enterocolitis (NEC) is an acute inflammatory necrosis of the bowel that primarily affects preterm infants and remains a leading cause of mortality and morbidity in neonatal intensive care units (NICU). Risk factors for classic NEC include prematurity, a feeding insult, abnormal bacterial flora, and intestinal ischemia/reperfusion injury with activation of proinflammatory cytokines [1]. While NEC has been recognized as a morbidity in mostly extremely preterm (EPT) neonates, a similar clinical condition occurs in term infants as well. “Term NEC” (infants >37-week gestational age) has also been studied in case series, with unique risk factors identified such as congenital left sided heart disease, hypoxic ischemic injury (perinatal asphyxia), polycythemia, and maternal drug abuse (especially cocaine) [2].

Most studies have focused on NEC in EPT infants <28-week gestational age (GA), since this group is at highest risk. Yet, it does occur in “older” preterm infants as well. NEC in moderately preterm (MPT) infants is reported to occur at a rate of ~1% [3, 4]-2.4%[5]. However, the total number of MPT births is much higher than EPT births: 9.5% vs 1.9% in the US (March of Dimes Peristats, 2013). Thus, a significant number of MPT infants are being diagnosed with NEC. It is plausible that this subset of older gestation preterm infants has unique risk factors and disease characteristics. Understanding these factors may help us identify infants at risk and prevent this devastating condition.

The purpose of our study was to describe NEC in MPT infants (28–34-week GA at birth). We hypothesized that, in comparison to the EPT group, MPT and late preterm/term (LP/T) infants who develop NEC would have a higher incidence of hemodynamic insults such as congenital heart disease, small for gestational age (SGA), pregnancy-induced hypertension (PIH)/preeclampsia, and perinatal asphyxia evidenced by the need for advanced resuscitation at birth. We also hypothesized that MPT infants who develop NEC will have a lower incidence of surgical NEC compared to EPT neonates.

## 2. Materials and Methods

This was a retrospective data analysis of all infants diagnosed with NEC that were admitted to a single tertiary care NICU over a period of 7 years from July 2009 to July 2016. The State University of New York at Buffalo Institutional Review Board (IRB) approval was obtained prior to data collection, and an exemption to individual parental consent was granted.

Infants were included with a diagnosis of NEC stage 2 or higher by Modified Bell's staging [6]. All the following criteria had to be met to confirm the diagnosis of NEC: (1) At least one abdominal radiograph with evidence of pneumatosis intestinalis reported by a pediatric radiologist; (2) clinical decision to treat with ≥ 7 days of antibiotics; and (3) withholding feeds for ≥ 7 days. Infants with incomplete maternal and birth data, especially those that were transferred in from other facilities with a diagnosis of NEC, were excluded. Other exclusions were infants with a diagnosis of NEC that was later ruled out in documentation by the attending physician and those without evidence of pneumatosis on imaging studies.

Subjects were divided into three groups based upon GA at birth: extremely preterm (EPT< 28 weeks), moderate preterm (MPT-28 to 33 6/7 weeks), and late preterm/term (LPT/T> 34 weeks). Differences between the groups were tested using Chi-squared test for categorical variables and ANOVA for continuous normally distributed variables. Results were considered significant when p < 0.05.

## 3. Results

### 3.1. Baseline Data

**Figure 1:**
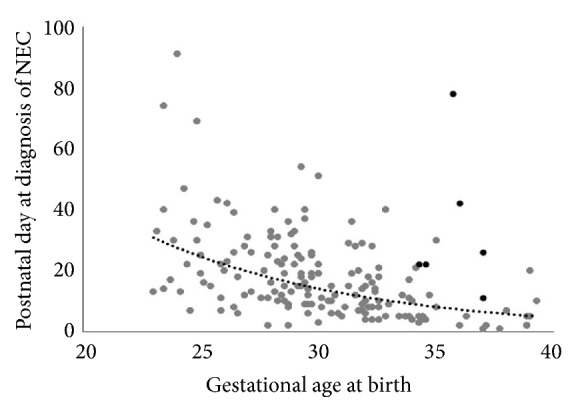
Inverse relationship between gestational age at birth and postnatal day at diagnosis of NEC. Each infant represented by a circle. Black circles represent the infants with congenital gastrointestinal anomalies (gastroschisis and Hirschsprung's disease).

Out of 6113 admissions to the NICU, 172 patients (2.8%) developed NEC (Table 1). Incidence of NEC decreased with increasing GA. The incidence of NEC in infants between 28 and 33^6^/_7_ weeks was 7%. There were more MPT infants with NEC (n=99) compared to the other two GA groups (n=73). There was an inverse relationship noted with postnatal age at presentation of NEC in relation to GA at birth (Figure 1), and most of the infants in our study group presented at an average GA of 29-33 weeks. A large percentage of infants over 35 weeks are not admitted to our NICU; hence, as a percentage of total hospital births >34 weeks, incidence of NEC in near term infants was 0.4% (30 infants with NEC in 7188 total live births >34-week GA in this study time period).

### 3.2. Risk Factors

The MPT and LPT/T cohorts with NEC had a significantly larger percentage of infants who were SGA (15% and 27% vs 4.6% in EPT) (Table 2). Additionally, a significantly higher number of infants born MPT were noted to be exposed to maternal hypertensive disorders than the EPT group and LPT/T group (24.4% vs 14% and 16%, resp.). There was no difference in the incidence of low APGAR scores at 5 min in the 3 groups, which was taken as in indicator of need for prolonged resuscitation.

### 3.3. Clinical Factors Prior to NEC

Presence of clinical factors that could have contributed to NEC was assessed among the three groups (Table 3). EPT infants with NEC had a higher incidence of hypotension soon after birth. Increased use of formula feeds as reflected by a mixed diet was noted in EPT and MPT compared to LPT/T. A significantly higher number of antibiotic days were noted in EPT infants prior to a diagnosis of NEC. The preterm cohorts also had a lower haematocrit, with more infants receiving a packed red cell (PRBC) transfusion prior to the diagnosis of NEC. Most congenital anomalies, gastrointestinal (GI) as well as genetic, were noted in MPT and LPT/T groups.

### 3.4. Outcome Characteristics

Exclusive involvement of the colon was more common, and need for surgery was less common with increasing gestation (Table 4). While not statistically significant, EPT infants showed a trend towards higher mortality from NEC.

## 4. Discussion

Necrotizing enterocolitis is the most common abdominal emergency of the preterm infant. We present this retrospective review to highlight the large number of MPT infants presenting with NEC and outline the morbidities associated with NEC.

Though GA is a continuum, different GA categories may have characteristic risk factors and clinical signs of disease. Over the last decade, mortality and morbidity among the MPT and LPT infants has received significant attention [7]. In currently available literature, there is little consensus on the definition of a MPT infant. Infants born between 28 and 34 weeks have been included in some studies, while others have focused on 32–34-week GA. Due to the high number of infants being delivered at this GA range, they constitute a significant healthcare burden. In a single center study, infants born at 28–34-week GA constitute ~37% of all NICU admissions and ~45% of NICU costs [8].

In our single center retrospective study of all inborn infants with stage 2 or greater NEC, we have noted an inverse relation between GA at birth and postnatal age at onset of disease (Figure 1). The EPT cohort (mean GA 25.5 weeks) developed NEC at ~25d, and the LPT/T cohort showed onset of disease much earlier (~13 d). This has been previously described in multiple epidemiological studies [9, 10], though the exact reason is not well understood. Possible reasons include inherent ontogeny of the vasculature development [11], change in microbiome [12], and age and gestation related changes in the inflammatory pathway such as toll-like receptor 4 (TLR-4) [13].

We noted a significantly higher percentage of MPT infants with NEC exposed to maternal hypertensive disorders compared to the other GA groups. Maternal hypertensive disorders, especially preeclampsia and eclampsia, are thought to predispose preterm infants to NEC. In a single center observational study, infants born to preeclamptic mothers had a higher incidence of NEC (22.9% vs 14.6%), earlier onset, more advanced stages of disease, and longer duration of illness as compared to infants born to normotensive mothers [14]. Contradictory findings have been demonstrated in several studies [15] including a large Swedish national health care analysis failing to show any significant correlation between preeclampsia and NEC [16]. A direct association cannot be determined from our findings as the majority of hypertensive disorders of pregnancy present only at term or late preterm, with just 10%–13% being diagnosed before 32 weeks [17].

In our study, MPT and LPT/T infants with NEC were more likely to be SGA. A retrospective study on infants with surgical NEC in Stockholm county also noted similar results with a higher incidence of intrauterine growth restriction (IUGR) in more mature infants (>28 weeks) [18]. IUGR is postulated to cause persistent hypoxia and ischemia by altering blood flow to the intestines [19]. Another retrospective study also found a higher risk of NEC in SGA neonates compared to appropriate for gestational age (AGA) neonates [20].

Another potential risk factor for NEC with similar pathophysiology is a hypoxic ischemic insult [21]. Coagulation necrosis and features specific to ischemic injury noted in intestinal samples of infants with NEC suggested ischemic injury as a major mechanism [22]. Rodent models of NEC were developed by an asphyxial insult in combination with enteral feeding [23]. In this study, we used APGAR scores to reflect the need for advanced resuscitation, in the absence of precise data on frequency of chest compressions and epinephrine use at delivery. Contrary to our hypothesis, MPT infants with NEC did not have an increased incidence of low APGAR scores compared to other age groups. As we did not analyse non-NEC infants, we cannot accurately assess if advanced resuscitation is in fact related to NEC or just a feature of immaturity. In a secondary analysis of MPT infants 29^0^/_7_ to 33^6^/_7_ weeks' GA enrolled in the Neonatal Research Network Moderate Preterm Registry, the rate of NEC progressively increased with the level of resuscitation; however, on logistic regression analysis after adjusting for covariates, the level of resuscitation did not statistically affect the incidence of NEC [24]. In a similar analysis of EPT infants, DeMauro et al. also showed a statistically insignificant relationship between observed rates of NEC and levels of resuscitation [25].

Chorioamnionitis and resulting inflammation of the fetoplacental unit are thought to contribute to neonatal morbidity. Recent studies have, however, failed to demonstrate an association of clinical or histological chorioamnionitis with NEC [26]. We did not find any difference in the incidence of reported chorioamnionitis in our study infants. In spite of this, EPT infants were noted to have a significantly higher number of antibiotic days prior to NEC. The frequent use of antibiotics postnatally in EPT infants is well described in the literature, with one study noting that 96% received early empirical antibiotics and 53% received more than 4 days of therapy [27]. High rate of initial antibiotic use is also related to the development of NEC, though attempting to establish this correlation was out of the scope of our study.

**Figure 2:**
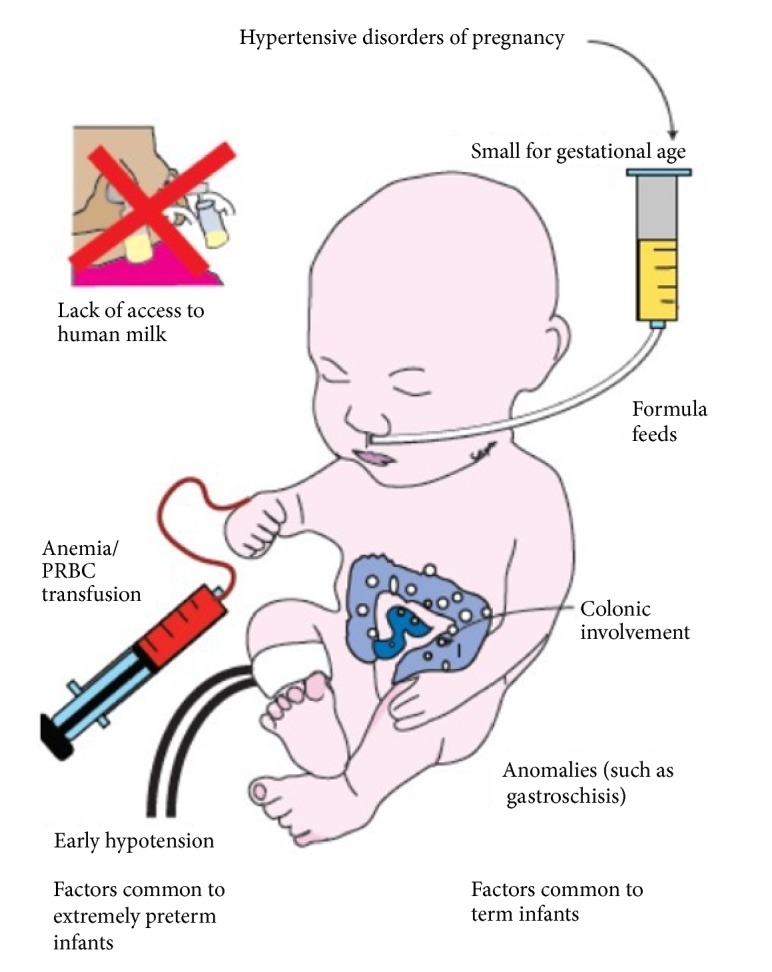
Salient risk factors for NEC noted in term and EPT infants are shown here. MPT infants share some characteristics of both groups, mostly related to a vascular etiology such as anaemia/transfusion and SGA status.

Discrete clinical characteristics of NEC in the MPT infants seemed to overlap with EPT groups. MPT infants were similar to EPT in receiving a mixed diet, being anaemic at onset of disease and having a history of PRBC transfusions just prior to diagnosis (Figure 2). Transfusion associated NEC or gut injury [28] has been described in anaemic preterm infants that are on a significant amount of enteral feeds [29]. A vascular etiology [30] has been postulated with alteration of the nitric oxide (NO) pathway; however, this entity remains controversial. The surprisingly high incidence of associated PRBC transfusions that we noted in the MPT group warrants further evaluation and monitoring of anaemia in this potentially lower risk population.

Infants with NEC in the MPT group also share some characteristics similar to LPT/T infants (presence of congenital anomalies, colonic NEC, and decreased need for surgical intervention). Exclusive involvement of the colon was not observed in EPT infants. Colonic involvement is more likely to present with bloody stools, and this finding has been described more commonly in > 28-week gestation infants with NEC than in extremely preterm infants [18]. Similar results were demonstrated in the moderate preterm NRN registry, with MPT more likely to be treated with medical treatment only vs EPT (1.7% vs 4% of all MPT and EPT infants) [5]. Mortality rates in this disease for term infants (4.7%) have also been reported to be less than premature infants (11.9%) [31].

## 5. Limitations

This is a retrospective data analysis of infants born at a single regional perinatal center. Only data recorded in the EMR could be collected. We do not have data on control infants at the same GA that did not develop this condition.

The incidence of NEC among MPT infants in our study is considerably higher than the previously reported data. Our institution is a Women and Children's Hospital with a large maternal transport service leading to a higher number of high-risk obstetric population including hypertensive disorders of pregnancy. However, we feel that the antecedent factors and clinical characteristics observed in our cohort are applicable to general population.

## 6. Conclusions

MPT infants who develop NEC may have risk and disease characteristics that overlap with both LPT/T and EPT groups. While reduced intestinal oxygen delivery (from hemodynamic factors such as hypotension or anaemia/transfusion) continues to be associated with NEC in MPT infants, they also tend to have associated congenital anomalies. Prenatal vascular factors (maternal hypertensive disorder and growth restriction) appear to play a major role in NEC in MPT infants. Recognition of these risk factors in this cohort may help in screening for NEC as well as in developing care practices for at risk infants.

## Figures and Tables

**Table 1:** Baseline characteristics.

Characteristic	EPT	MPT	LPT/T
Incidence of NEC as % of NICU admits at corresponding GA	10% (43/399)	7% (99/1359)	0.7% (30/4355)
GA at birth (weeks, mean ± SD)	25.5±1.4	30.5±1.7	36.1±1.8
Birth weight (kg)	0.77±0.18	1.41±0.37	2.54±0.6
Postnatal day at onset of NEC (d)	25.9±18	18±15	12.7±16.2
Postmenstrual age at diagnosis (weeks)	29.2±2.4	33.1±2.2	37.9±2.9

**Table 2:** Ante and Perinatal risk factors for NEC.

Characteristic	EPT (n=43)	MPT (n=99)	LPT/T (n=30)	P value
Chorioamnionitis	5 (11.6%)	7 (7)	1 (3.3%)	0.4
Maternal hypertensive disorders	5 (11.6%)	29(29%)	5 (16%)	0.04*∗*
5 min APGAR score ≤ 5	3 (6.9%)	2 (2%)	1 (3.3%)	NS
Small for gestational age	2 (4.6%)	15 (15%)	8 (27%)	0.02*∗*

**Table 3:** Clinical factors prior to diagnosis of NEC.

Characteristic	EPT (n=43)	MPT (n=99)	LPT/T (n=30)	P value
Hypotension in first 24 hrs after birth	9 (20.9%)	4 (4%)	0	0.0005*∗*
Exclusive breast milk	2 (4.6%)	1(1%)	2 (6.6%)	NS
Mixed Diet (formula and breast milk)	32 (74.4%)	76 (76.8%)	16 (53.4%)	0.04*∗*
Exclusive formula diet	9 (21%)	23 (23%)	12 (40%)	NS
Antibiotic days prior to NEC	7.7±4.5	4.9±3.5	5.8±3.5	0.002*∗*
Hematocrit at diagnosis (%)	33.5±5.6	34.5±6.3	41.8±10.4	0.005*∗*
Transfusion in the 48hrs prior to diagnosis	11 (25.6%)	12 (12.1%)	0%	0.02*∗*
Congenital Gastrointestinal abnormalities†	0	7 (7%)	6 (20%)	0.006*∗*
Genetic/congenital non GI abnormalities‡	0	5 (5%)	3 (10%)	0.13

† Congenital GI anomalies included gastroschisis (4), Hirschsprung's disease, imperforate anus, jejunal atresia and Meckels diverticulum. ‡ Genetic/non GI abnormalities included Trisomy 13, Trisomy 21, congenital myopathy, meningomyelocele and 17q21 deletion.

**Table 4:** Outcome characteristics.

Characteristic	EPT (n=43)	MPT (n=99)	LPT/T (n=30)	P value
Colonic NEC	0	19 (19.19%)	10 (30.03%)	0.0006*∗*
Surgical NEC	22 (53%)	20 (21%)	1 (3.3%)	0.0031*∗*
Death before discharge	8 (18.6%)	9 (9%)	1 (3.3%)	0.08

## Data Availability

The data used to support the findings of this study are included within the article.
